# The identification of age-associated cancer markers by an integrative analysis of dynamic DNA methylation changes

**DOI:** 10.1038/srep22722

**Published:** 2016-03-07

**Authors:** Yihan Wang, Jingyu Zhang, Xingjun Xiao, Hongbo Liu, Fang Wang, Song Li, Yanhua Wen, Yanjun Wei, Jianzhong Su, Yunming Zhang, Yan Zhang

**Affiliations:** 1 College of Bioinformatics Science and Technology, Harbin Medical University, Harbin, 150081, China; 2 Department of Gerontology, The First Affiliated Hospital of Harbin Medical University, Harbin 150001, China; 3 Department of Neurology, The Second Affiliated Hospital of Harbin Medical University, 246 Xuefu Road, Harbin 150086, China

## Abstract

As one of the most widely studied epigenetic modifications, DNA methylation has an important influence on human traits and cancers. Dynamic variations in DNA methylation have been reported in malignant neoplasm and aging; however, the mechanisms remain poorly understood. By constructing an age-associated and cancer-related weighted network (ACWN) based on the correlation of the methylation level and the protein-protein interaction, we found that DNA methylation changes associated with age were closely related to the occurrence of cancer. Additional analysis of 102 module genes mined from the ACWN revealed discrimination based on two main patterns. One pattern involved methylation levels that increased with aging and were higher in cancer patients compared with normal controls (HH pattern). The other pattern involved methylation levels that decreased with aging and were lower in cancer compared with normal (LL pattern). Upon incorporation with gene expression levels, 25 genes were filtered based on negative regulation by DNA methylation. These genes were regarded as potential cancer risk markers that were influenced by age in the process of carcinogenesis. Our results will facilitate further studies regarding the impact of the epigenetic effects of aging on diseases and will aid in the development of tailored cancer preventive strategies.

Aging is an intricate and universal physiological process that is affected by many factors, such as telomere shortening, the accumulation of genetic mutations, oxidative stress, and the decay of cells and organs^1^. Aging gradually reduces the body’s ability to maintain a steady state and increases the risk of disease, including cancer, cardiovascular diseases and neurodegenerative diseases. However, according to the limited genetic backdrop, the human aging phenomenon is not completely understood. The emergence of epigenetics provides a new direction for solving this problem. DNA methylation is a widely studied epigenetic modification that has an important influence on human traits and cancers. Many studies have revealed that CpG methylation plays an important role in maintaining gene silencing, and this process is essential for a wide variety of cellular functions, such as tissue-specific and development-specific gene expression, X chromosome inactivation and genomic imprinting^2^,^3^,^4^.

Numerous studies have focused on the relationship of epigenome state and age, especially DNA methylation and chronological age, which spans a wide range^5^,^6^. Heyn *et al.*^7^ performed genome-wide bisulfite sequencing for newborns and centenarians, revealing that centenarians exhibit lower DNA methylation levels and that the correlation between neighbor CpGs is weaker throughout the entire genome of centenarians compared with newborns. Age-related CpG sites have been identified in numerous studies^8^,^9^,^10^,^11^, and these DNA methylation changes are significantly enriched in the region of the bivalent chromatin promoter^12^ and Polycomb group protein target genes^13^. Various age-dependent hypermethylation CpGs are enriched with DNA binding factors and transcription factors, indicating that the dysregulation of these so called master genes can affect a large number of biological processes^14^. Thus, these age-dependent hypermethylation CpGs can explain a large portion of the phenotypic variation observed with aging^11^. In addition, age-related CpGs primarily exhibit linear DNA methylation changes during aging; thus, they can serve as a predictor of age^11^,^15^,^16^. Horvath found an epigenetic clock by identifying human biological age markers and accurately predicted age based on DNA methylation^5^. In another study, Weidner *et al.* demonstrated that only three age-related CpGs can be used to estimate biological age in blood^17^. Different aging rates can partly explain epigenetic drift, and the discovery of an increased aging rate in tumor tissues may provide a quantitative strategy for studying DNA methylation effects on age-associated diseases^18^.

The occurrence of tumors has been primarily described with regard to genome mutations, but numerous recent researches have demonstrated that aberrant DNA methylation plays a fundamental role in cancer^19^,^20^,^21^,^22^. Simultaneously, tumor suppressor gene promoter hypermethylation is noted in tumor cells. However, gene promoter hypermethylation is also noted in normal non-proliferative cells as age increases. So *et al.* studied tumor suppressor genes in normal gastric epithelial cells and found that *LOX*, *p16*, *RUNX3* and *TIG1* promoter methylation is highly correlated with age. This finding demonstrates that tumor suppressor gene promoter methylation elevates tumor susceptibility in aging populations^23^.

Researchers have gradually realized that the process of aging and cancer have similar foundations rooted in epigenetics^24^. The research on the mechanism of aging epigenetics has suggested that peripheral blood DNA methylation is closely related to aging^12^. Aberrant DNA methylation is a common character in numerous diseases, including cancer. Global hypomethylation and site-specific promoter CpG island hypermethylation were previously described in the cancer epigenome^21^. Similarly, various tumor suppressor genes hypermethylated in cancer also undergo *do novo* methylation in normal tissues. For example, various genes that regulate development exhibit increased methylation during the aging process, whereas other genes involved in the defense and immune response exhibit loss of methylation with age^8^. Cruickshanks *et al.* used whole-genome bisulfite sequencing to demonstrate that the progress of malignancy may be due to DNA methylation of senescence cells if these cells bypass the switch that prevents their growth^25^. This study indicates that senescence cells are more prone to cancer and provides a novel idea to develop cancer therapies.

Network construction is an effective and reliable method for integrating complicated biological correlations and has successfully been applied to identify biomarkers^26^,^27^. Herein, by analyzing DNA methylation profiles of healthy aging populations and multi-type cancer patients and controls, we constructed an integrated age-associated and cancer-related protein interaction weighted network. Then, based on changing DNA methylation patterns (HH pattern and LL pattern) in aging and cancer, we identified 25 age-associated cancer markers. Moreover, *HAND2*, *GDNF* and *ALX1* were associated with the prognosis of kidney renal clear cell carcinoma. Our analysis revealed that dynamic DNA methylation changes in aging may be associated with carcinogenesis.

## Results

### Identification of age-associated differentially methylated genes (aDMGs) at the genome scale

We downloaded DNA methylation profiles (Illumina Infinium HumanMethylation27 BeadChip) based on peripheral blood samples from 394 healthy Dutch individuals 16 to 88 years of age. During the preprocessing procedure, the CpG sites with missing values in the profile were discarded. Then, 23727 CpG sites that mapped to 12175 genes (see the Materials and Methods) were available for further analysis. Given that age-related DNA methylation changes were primarily inclined to linear approximation^11^,^15^,^16^, age-associated gene was predicted using a linear correlation model for each gene in the above individuals. Using 1000 random perturbations with a FDR threshold of 0.01, we identified 4277 genes significantly associated with age, including 1614 genes wherein methylation increases with advancing age (defined as hyper-aDMGs) and 2663 genes wherein methylation decreases with age (defined as hypo-aDMGs). We compared our aDMGs with known age-associated genes in the GenAge database^28^, a database of genes related to human aging that includes greater than 298 entries. We identified 86 known genes in our study, such as *WRN, XRCC5, p16*, etc. The *WRN* gene encodes a helicase and exonuclease involved in numerous DNA repair and processing pathways; this gene is one of the strongest candidate genes influencing human aging in GenAge. The *XRCC5* gene is associated with telomeres and *WRN*.

**Figure 1:**
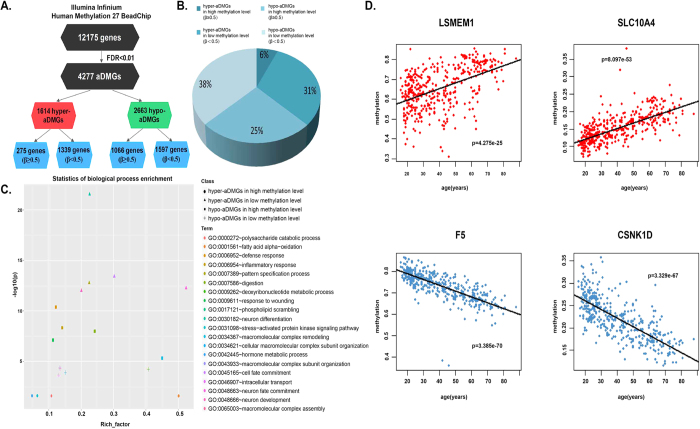
Age-associated differentially methylated genes (aDMGs) identified in human whole blood samples. (**A**) Identification of age-associated differentially methylated genes (aDMGs) from Illumina Infinium HumanMethylation27 BeadChip. Of 4277 aDMGs included in the analysis, 1614 were methylated increasing with age (hyper-aDMGs), and 2663 genes were methylated decreasing with age (hypo-aDMGs). (**B**) The further classification for hyper-aDMGs and hypo-aDMGs, and the proportion of each category. (**C**) Scatterplot for statistics of biological process enrichment. For each category of aDMGs, we listed the top five enriched biological processes. Rich_factor is the ratio of the number of aDMGs mapped to this GO term and the number of annotated genes in this term. The higher rich factor means the more significant enrichment. The higher -log10 (p) also means the more significant enrichment, where p is the p-value for aDMGs enriched GO term. (**D**) Illustrate the four categories of aDMGs.

These aDMGs were divided into two classes based on DNA methylation level: high methylation level and low methylation level. A gene was defined as high methylation level if the average methylation level in all samples was at least 0.5 (β ≥ 0.5). The cutoff value of methylation level was refered to Zhang *et al.*^29^. Similarly, a low methylation level indicates that the average methylation level of a gene was less than 0.5 (β < 0.5). In total, the hyper-aDMGs group consisted of 275 genes with high methylation level and 1339 genes with low methylation level. The hypo-aDMGs consisted of 1066 genes with high methylation level and 1597 genes with low methylation level (Fig. 1A,B,D). The proportion implied that the methylation changes (increase versus decrease) as age increase are affected by the gene’s initial methylation status. Hyper-aDMGs are more likely to exhibit low methylation level, whereas hypo-aDMGs are more likely to exhibit high methylation level (odds ratio = 3.25; 95% confidence interval [CI] = 2.79 to 3.79; Fisher’s Exact Test p = 1.41e-58).

Gene ontology functional enrichment analysis revealed that these aDMGs were enriched in several biologically relevant ontologies. Hyper-aDMGs were enriched in differentiation and developmental processes, whereas hypo-aDMGs were enriched in immune response and defense response processes. These results indicate the different roles of these gene categories in aging (Supplementary Table S1). We analyzed the statistics of biological process enrichment for four categories genes in detail (Fig. 1C). Hyper-aDMGs with low methylation level exhibited greater enrichment both on rich factor and p-value compared with other groups. These genes were primarily involved in neuron differentiation and fate commitment. In contrast, hyper-aDMGs with high methylation level were enriched in the stress-activated protein kinase signaling pathway and hormone metabolic processes. Hypo-aDMGs with high methylation level exhibited more significant enrichment compared with hypo-aDMGs with low methylation level. These genes were likely to participate in defense responses and inflammatory responses. Hypo-aDMGs with low methylation level were enriched in the biological processes of macromolecular complex subunit organization and intracellular transport. It appeared that hyper-aDMGs with low methylation level and hypo-aDMGs with high methylation level exhibited paramount functions in aging.

**Figure 2:**
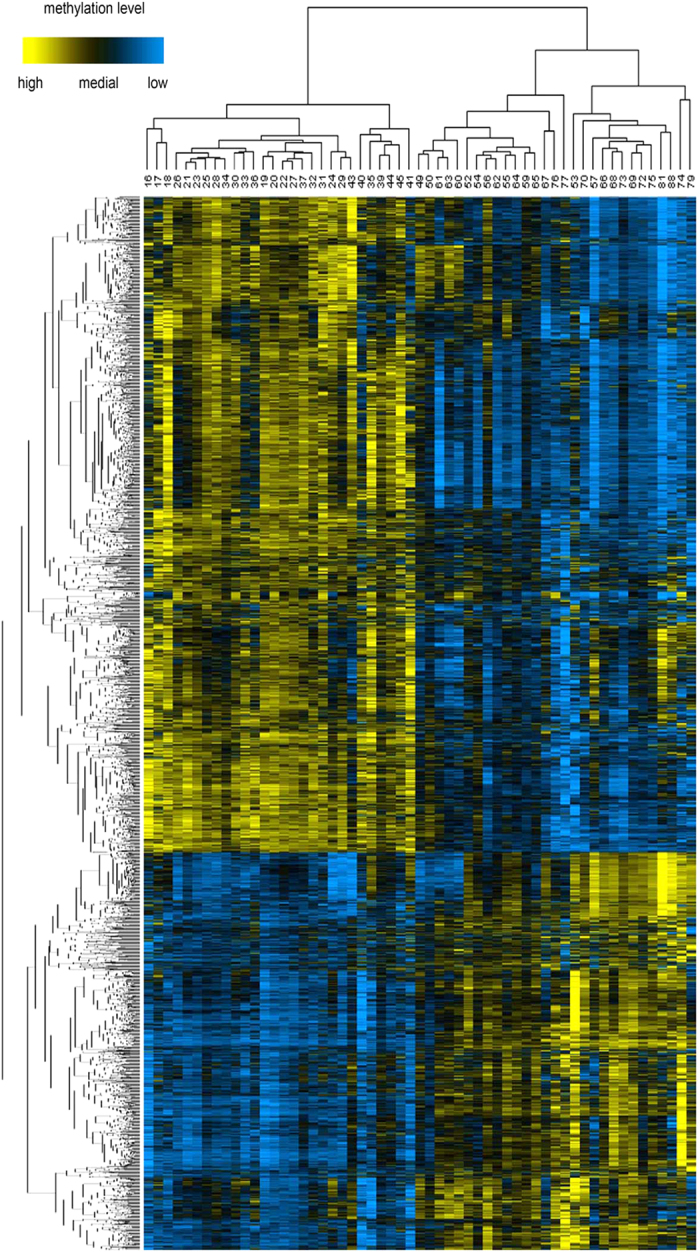
Hierarchical clustering of age-associated differentially methylated genes. Rows represent genes and columns represent samples. For a gene, yellow represents the higher methylation level, blue represents the lower methylation level and black represents the medial methylation level for all samples.

Furthermore, we performed hierarchical clustering analysis for samples and genes based on Pearson correlation to investigate aDMG variations in aging. Here, to decrease errors among samples and expediently observe the results, we performed the hierarchical clustering analysis by using the mean methylation of a group of samples with the same age. The number of unique ages among the 394 individuals considered was 57, thus we clustered 57 samples instead of 394 samples (Fig. 2). These samples were classified into two groups by a cutoff of 50 years of age (with the exception of a 49-year-old sample in the right portion). The result was consistent with the announcement by the United Nations Educational Scientific and Cultural Organization (UNESCO) that suggests that a man is classified as middle-aged after 50 years of age. From the view of epigenetics, obvious changes in the methylation status of promoter CpG sites were noted in our study. This observation might be the result of gradual molecular changes in the body. The aged population is at high risk for cancers given that carcinogenic factors accumulate to a certain extent during aging.

### Identification of cancer differentially methylated genes (cDMGs)

Next, we used QDMR^30^ based on entropy to identify genes that are differentially methylated between tumor samples and normal samples of each cancer (see the Materials and Methods). As a consequence, we identified a set of genes for each cancer type. We referred to these genes as cancer differentially methylated genes (cDMGs), and this group included 4452 genes. GO biological process enrichment analysis for these cDMGs was performed using DAVID. The results indicate that these genes are involved in many cancer related processes, such as homeostatic processes, defense response, biological adhesion, and ion transport (Supplementary Fig. S1). Moreover, we further addressed the relationship between cDMGs identified by us and bivalently marked and ploycomb group target genes (PCGTs). We collected 3257 human bivalent genes from BGDB database^31^ and 1861 PCGTs from one study^32^. The set of 14495 genes located in HumanMethylation27 BeadChip was used as a background set, hypergeometric tests were performed to test whether the cDMGs were enriched for bivalently marked and polycomb group target genes. Among the cDMGs, 1279 (39.27%) were bivalent genes and 850 were (45.67%) PCGTs. The results manifested that the cDMGs identified in this study were strongly enriched for bivalently marked genes (p < 0.001) and PCGTs (p < 0.001), implying it was consistent with previous published literatures^33^. Additionally, some of these cDMGs are related to cancer as noted in PubMeth^34^. For example, *TMEFF2* exhibited increased DNA methylation levels in BRCA, COAD, LUAD and LUSC compared with corresponding normal samples in our study. Previous studies indicated that this transmembrane protein with a single EGF-like and two follistatin domains (*TMEFF2*) was epigenetically silenced in numerous tumor types, suggesting a potential role as a tumor suppressor^35^,^36^. Thus, the method used to identify differentially methylated genes is effective.

There were 1668 cDMGs overlapped with aDMGs, 2784 genes only differentially methylated in cancer and 2609 genes only differentially methylated in aging (Supplementary Fig. S2). GO biological process enrichment analysis was performed for the three categories of genes using DAVID, respectively. All significant (Bonferroni adjusted p < 0.05) terms of genes only differentially methylated in aging, and top ten significant terms of genes only differentially methylated in cancer as well as overlapped genes were listed in Supplementary Fig. S3. Genes only differentially methylated in aging were mainly enriched in Golgi vesicle transport and regulation of cellular metabolic process. Genes only differentially methylated in cancer were enriched in immune response, defense response and response to wounding. While overlapped genes were primarily involved in cell-cell signaling, cell adhesion and neuron differentiation, etc. The results implied that some age-associated genes might be involved in the carcinogenesis and some might not.

### Construction of an age-associated and cancer-related weighted network

In recent decades, the construction of biological networks has become an essential method in systems biology research. The weighted graph provides a richer description since it considers the topology along with the quantitative information on the dynamics occurring in the whole network^37^. Hence, given that the complex correlation between aging and cancer, we constructed an age-associated and cancer-related weighted network (ACWN).The ACWN was constructed through mapping aDMGs and cDMGs step by step in this study for two considerations. Firstly, we want to examine whether DNA methylation changes associated with age are closely related to cancer. Secondly, we want to further detect which age-associated genes and what the DNA methylation patterns they present will be more inclined to affect cancer.

In order to achieve these targets, we mapped age-associated genes into PPI to construct an age-associated network in the first place instead of treating them equally. 4277 aDMGs were mapped to a human protein-protein interaction network (PPI) that contained 14611 genes and 80496 interactions^26^. Then, an age-associated weighted network (ASWN) was obtained by extracting aDMG pairs and the interactions among these pairs. The aDMGs were used as nodes in the ASWN, and the Pearson correlation coefficients of the DNA methylation status of each aDMG pair were used as linkage weights. In ASWN, the weights are used to indicate the strength of co-methylation between gene pairs. If the genes in one pair are interacted and co-methylation, they may be involved in similar regulation mechanisms as well as similar functions. Thus, we considered that the weighted network can more reasonably reflect the functional structure of biological network. In order to obtain a robust age-associated network, we removed the edges with weak correlation by the weighting threshold (permutation test, p < 0.01). Finally, we extracted a closely age-related gene interaction network based on DNA methylation correlations; the network consisted of 2200 nodes and 4583 edges (Supplementary Fig. S4).

**Figure 3:**
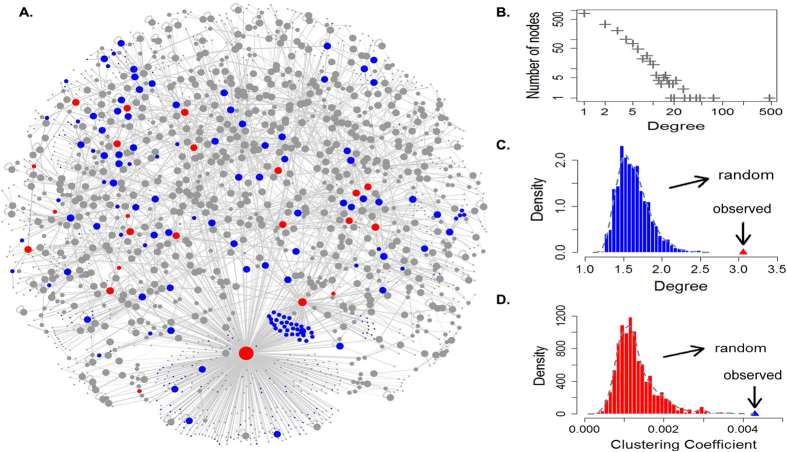
An age-associated and cancer-related weighted network. (**A**) Red nodes show the genes are at least in two modules, and blue nodes show the genes are only in one module. While grey nodes show the genes are not in any module. (**B-D**) Network topological features and the comparison between real network and random networks. The degree distribution in ACWN follows a power-low distribution. And both average degree and average clustering coefficient of ACWN are higher than random networks (1000 random networks).

Next, to address whether DNA methylation changes associated with age are closely related to cancer, an age-associated and cancer-related weighted network (ACWN) including 1697 nodes and 2595 edges was extracted from ASWN by mapping into cDMGs (Fig. 3A). The ACWN contained the cDMGs and the genes that were directly connected with cDMGs in ASWN. Thus, the nodes in ACWN were differential either only in aging (aDMGs) or both in aging and cancer (acDMGs). Then the topological features of ACWN were computed. The average degree and clustering coefficient were 3.06 and 0.043, respectively. The degree of ACWN followed a power-low distribution indicated that the network was a scale-free network in which most nodes had a low degree and only a few nodes with high connectivity (Fig. 3B). To demonstrate that the ACWN exhibited specific epigenetic relations between genes in aging and cancers, we compared topological features of the ACWN and random networks. The average degree and average clustering coefficient of the ACWN were both higher compared with random networks (Fig. 3C,D). These results implied that the ACWN was a densely connected network community. Moreover, the DNA methylation changes between age-associated genes and carcinogenesis genes were not random and independent events. Thus, not surprisingly, cancers frequently occur during the process of aging.

However, not all age-associated genes were involved in cancer. Hence, our study aimed to furtherly detect age-associated genes and in which the DNA methylation patterns they present were more inclined to affect cancer. Consequently, we compared cancer-related aDMGs (genes in ACWN) and cancer-unrelated aDMGs (genes not in ACWN) in ASWN to determine the difference between the two types of aDMGs. The ASWN contained 1697 cancer-related aDMGs and 503 cancer-unrelated aDMGs. First, we compared the topological characters of the two types of aDMGs in ASWN. The average degrees of cancer-related aDMGs and cancer-unrelated aDMGs were 4.68 and 2.43, respectively, and the average clustering coefficients of cancer-related aDMGs and cancer-unrelated aDMGs were 0.067 and 0.031, respectively. The results may indicate that cancer-related aDMGs were more likely to be located in the topological center of ASWN, while cancer-unrelated aDMGs were mainly located in the topological periphery. Next, we compared the proportions of aDMGs in the four categories to discover differences in DNA methylation patterns (Supplemental Table S2). The cancer-related and cancer-unrelated aDMGs shared the same proportion of hyper-aDMGs with high methylation level. Greater than half of cancer-unrelated aDMGs (52.09%) were categorized as hypo-aDMGs with low methylation level, whereas the proportion decreased to 37.01% for cancer-related aDMGs. In contrast, the proportions of cancer-related aDMGs categorized as hyper-aDMGs with low methylation level and hypo-aDMGs with high methylation level both increased compared with cancer-unrelated aDMGs. Thus, these two categories of aDMGs were involved in more crucial functions as previously mentioned. Finally, we compared the functional differences between cancer-related aDMGs and cancer-unrelated aDMGs. The cancer-related genes were involved in a multitude of aging and cancer related biological processes, such as regulation of response to external stimulus, regulation of cell proliferation and cell adhesion. The cancer-unrelated genes were enriched in DNA metabolic process, DNA replication and the regulation of transcription from RNA polymerase II promoters. In conclusion, we discussed the differences between cancer-related and cancer-unrelated aDMGs that may provide an explanation for why some age-related genes are involved in cancer and some are not.

### Detecting modules of associated with age and cancer in ACWN

**Figure 4:**
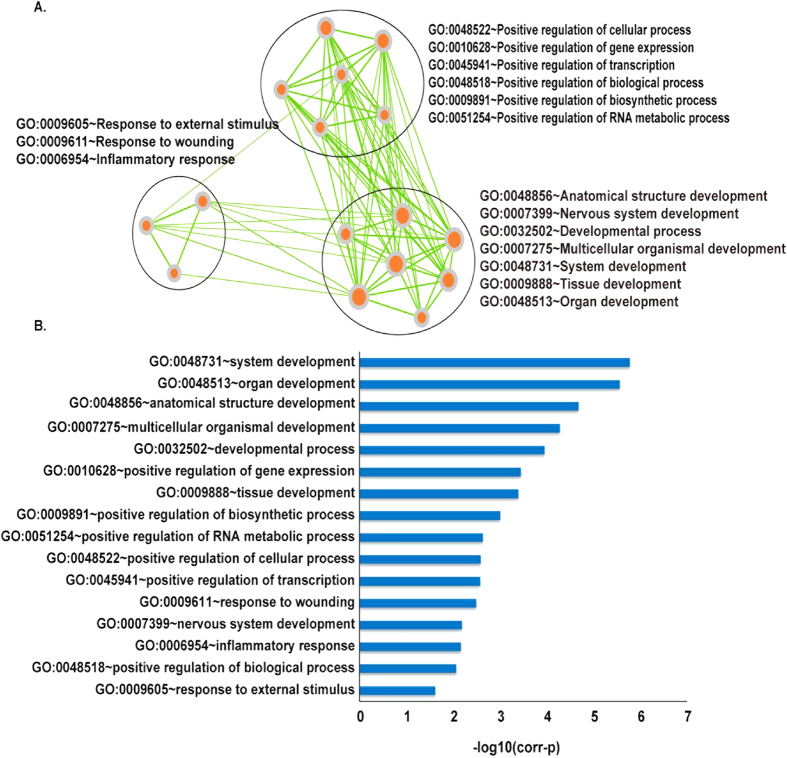
DAVID enrichment analysis for genes in the modules. (**A**) The nodes represent the GO_BP terms in the enrichment analysis using DAVID. The size of a node represents the gene count in this term. Three groups were formed by Enrichment Map plugin in Cytoscape. (**B**) Corr-p in X axis is Bonferroni adjusted p value.

Furthermore, we hypothesized that the DNA methylation changes associated with age affected cancer risk by clustered in the human interactome. Thus, a Cluster ONE (Clustering with Overlapping Neighborhood Expansion) algorithm^38^ was used to detect significant modules in the ACWN. The Cluster ONE algorithm is a graph clustering algorithm that can handle weighted graphs and readily generate overlapping clusters. In total, we obtained 31 modules including 238 genes (Supplementary Table S3). Of these genes, 48 genes overlapped with hub genes that defined as the top 15% of the nodes by degree, and 186 genes were directly connected with hubs. The hubs are important to the stability of the biological system and form a central part of the network. Likewise, almost module genes showed prominent positions in the network, implying that these genes may play critical roles. Moreover, function enrichment analysis of module genes in biological process revealed enrichment in several development processes, the regulation of biological processes and response to external stimulus (Fig. 4A,B, Table 1). *HNF4A* is a nuclear transcription factor that controls the expression of several genes, and this gene exhibits the largest degree in ACWN. This gene may play a role in development of the liver, kidney, and intestines. Neuregulin-1 (*NRG1*) interacts with a hub gene, and it is an overlap gene involved in a number of modules. *NRG1* plays a critical role in the growth and development of multiple organ systems. Dysregulation of this gene is associated with diseases, such as cancer. A previous study demonstrated that tumor suppressor *NRG1* is frequently subject to epigenetic silencing in epithelial tumors^39^. Berman *et al.* also showed *NRG1* had hypermethylated promoters and reduced expression in colorectal cancer which consistent with our results^40^. *KDR* is one of the two receptors of *VEGF*, which is a major growth factor for endothelial cells. Kim *et al.* reported that *KDR* promoter hypermethylation is correlated with decreased expression in stomach cancer, colon cancer and hepatocellular carcinoma^41^. The epigenetic silencing of *KDR* should be considered in the activation of the *VEGF-VEGFR* signaling pathway in the cancer cells. In addition, the vascular endothelial growth factors receptors *KDR* (*VEGF-R2*) and *FLT4* (*VEGF-R3*) are important regulators of angiogenesis and lymphangiogenesis, and these genes interact in one module we identified. Quentmeier *et al.* demonstrated that *KDR* and *FLT4* were epigenetically regulated genes that can be silenced by methylation in human umbilical vein endothelial cells^42^. Jesmin *et al.* examined age-related changes in cardiac expression of *VEGF* and its angiogenic receptor *KDR* in stroke-prone spontaneously hypertensive rats^43^.

Moreover, we also demonstrated that hyper-aDMGs in the modules were more likely to exhibit a low methylation level and hypo-aDMGs were likely to exhibit a high methylation level. This result is consistent with the tendencies observed for total aDMGs as previously described. However, the genes of the two classes in the modules accounted for a higher proportion (81.51%, 194/238) than that in total aDMGs (56.23%, 2405/4277) (Chi-square test, p-value = 2.675e-14, Supplementary Fig. S5). Given that some genes in the modules were not only aDMGs but also cDMGs (acDMGs), we hypothesized that cancer-related differential methylation may likely arise in these two conditions, thus providing one plausible explanation for this inconsistent proportion. These genes generally share a similar epigenetic foundation in aging and cancer: hyper-aDMGs exhibited increased DNA methylation in tumor tissues compared with normal controls, whereas hypo-aDMGs exhibited reduced DNA methylation in tumor tissues compared with normal controls. Yuan *et al.* previously demonstrated that genes undergoing age-associated hypermethylation tended to show increased methylation in cancer compared with normal tissue and that sites undergoing age-associated hypomethylation exhibited corresponding hypomethylation in cancer tissues compared with normal tissues^14^. In summary, understanding the trends of methylation changing in aging (hyper-aDMGs or hypo-aDMGs), methylation level (high or low) and differential direction in tumor and normal tissues (differentially hypermethylated or hypomethylated in cancer than normal) is biologically and clinically important.

### Identification of age-associated cancer markers combined with gene expression

**Figure 5:**
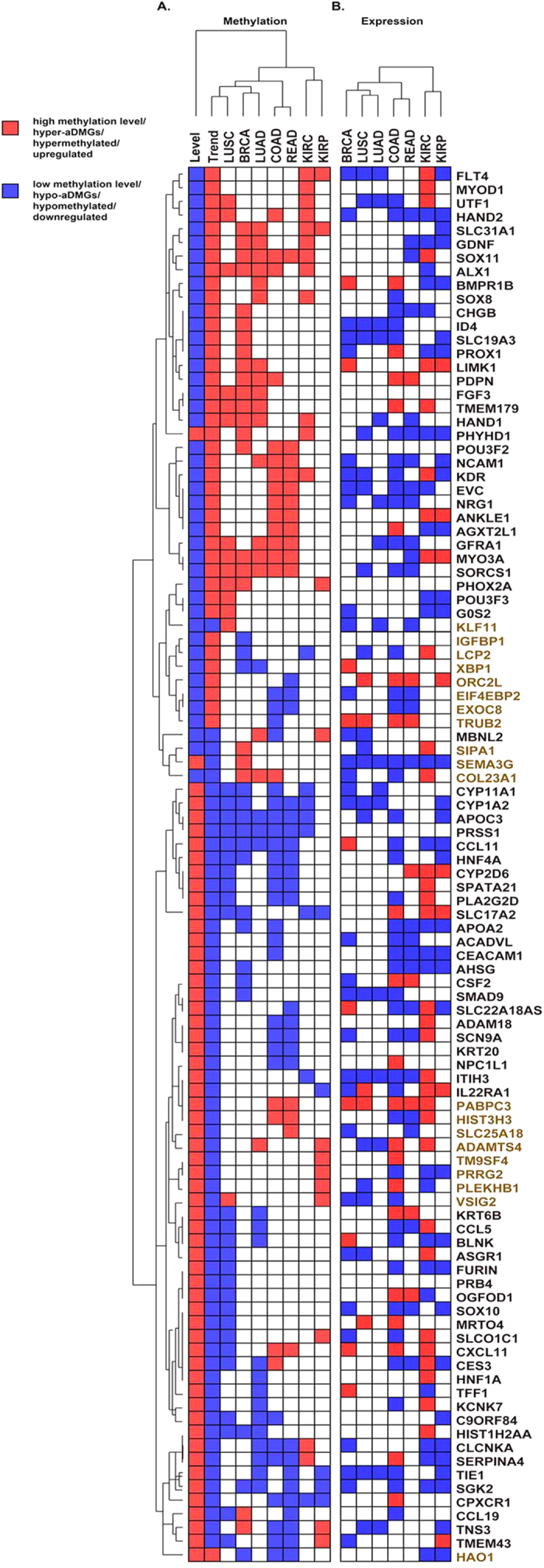
DNA methylation and gene expression pattern mappings in aging and cancer. (**A**) DNA methylation pattern maps of 102 acDMGs in the ACWN. In first column “Level”, the red block means the gene exhibits high methylation level (β ≥ 0.5), and the blue one means the gene exhibits low methylation level (β < 0.5). In the second column “Trend”, the red block means the methylation level of a gene is increasing with advancing age, and the blue one means the methylation level of a gene is decreasing with advancing age. In the rest of columns, red blocks represent the genes have higher methylation in cancer than normal, and blue ones represent the genes have lower methylation in cancer than normal. (**B**) Similarly, red blocks represent the expression levels of genes are upregulated and blue ones represent the expression levels of genes are downregulated.

Therefore, an integrated DNA methylation pattern mapping was established by focusing on 102 acDMGs among 238 module genes, and we observed an interesting phenomenon (Fig. 5A). As expect, acDMGs mainly showed two types of patterns. First, hyper-aDMGs exhibited increased methylation in cancer tissues compared with normal tissues; we defined this pattern as the HH pattern. Second, hypo-aDMGs exhibited reduced methylation in cancer tissues compared with normal controls, and we defined this pattern as the LL pattern. Among 102 genes, 82 genes (except *KLF11*, *IGFBP1*, *LCP2, XBP1*, *ORC2L*, *EIF4EBP2*, *EXOC8*, *TRUB2*, *SIPA1*, *SEMA3G*, *COL23A1*, *PABPC3*, *HIST3H3*, *SLC25A18*, *ADAMTS4*, *TM9SF4*, *PRRG2*, *PLEKHB1*, *VSIG2*, and *HAO1*) exhibited changes in methylation in aging and cancer that were consistent with the two patterns we defined. HH pattern genes generally showed low methylation level, whereas LL pattern genes mainly showed high methylation level. We inferred genes in HH pattern may function as tumor suppressor genes in cancer, while genes in LL pattern may function as an oncogene. Besides, the directions of differential methylation of these acDMGs noted between cancer and normal tissues from different cancers were generally consistent, indicating that the acDMGs we identified appeared to be widespread in carcinogenesis and normal aging processes. In particular, we observed that colon carcinoma (COAD, 11), rectum adenocarcinoma (READ, 8) and kidney renal clear cell carcinoma (KIRC, 8) exhibited more age-associated genes compared with other cancers, suggesting that aging was more likely to be one of the earliest changes marking the risk for neoplasia in these cancers. In addition, similar cancers were clustered together by acDMGs.

Given that DNA methylation is implicated in the regulation of gene expression, we investigated the expression levels of 82 genes in cancer and normal samples. The differential expression directions of these genes in different cancers were inconsistent and relatively disordered compared with the differential methylation results (Fig. 5A,B). Combining the changes of DNA methylation and gene expression, we identified 28 genes as age-associated cancer markers exhibiting promoter hypermethylation and transcriptional silencing or promoter hypomethylation and transcriptional activation. Nineteen genes were classified as the HH pattern, and nine genes exhibited the LL pattern. Functional annotation analysis revealed that HH pattern markers were mainly annotated in many development processes and the regulation of transcription, whereas LL pattern genes mainly participated in multiple metabolic processes, transport and homeostasis processes (Supplementary Table S4). Most markers were specific to one cancer type, whereas others were age-associated multi-cancer markers.

### Validation of age-associated cancer markers

**Figure 6:**
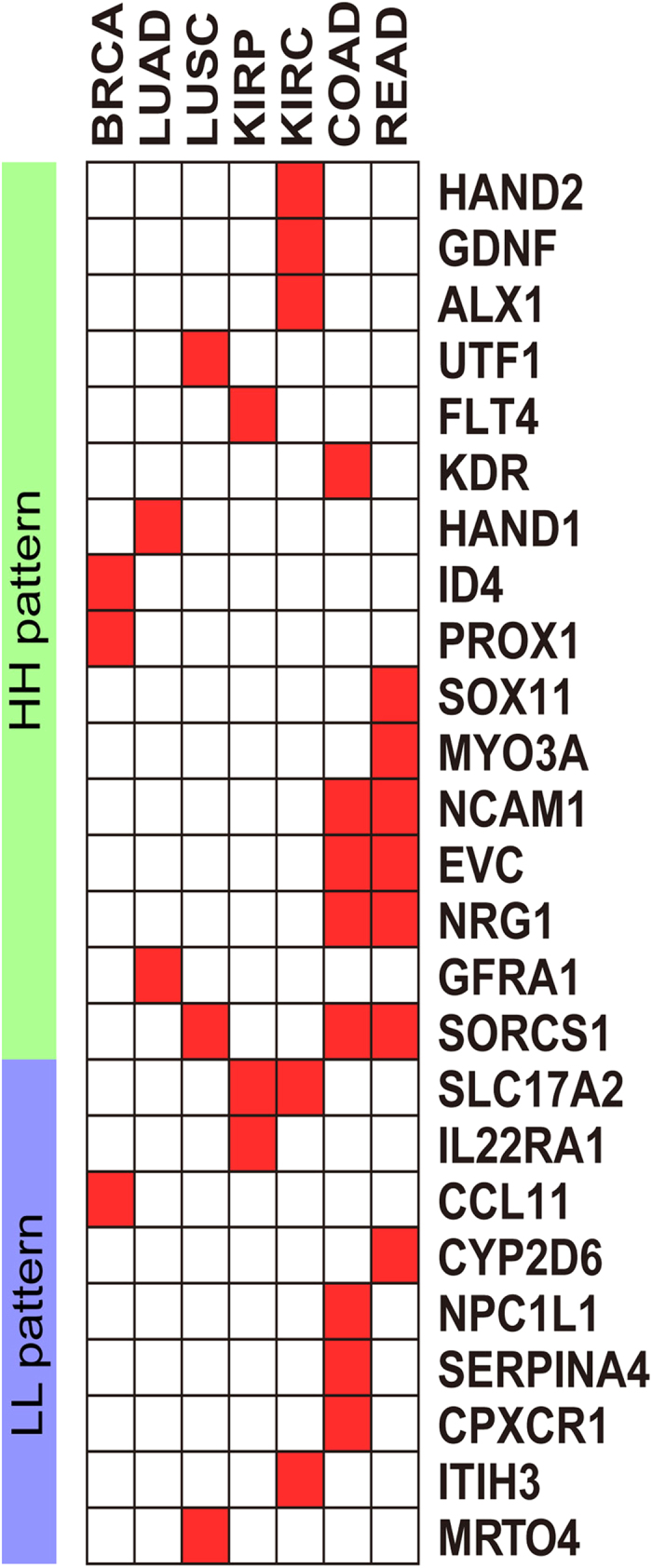
Age-associated cancer markers. Red blocks represent that the genes are age-associated cancer markers identified in the corresponding cancer.

In order to validate the reliability of these 28 markers further, we applied our method to all the other HumanMethylation27 BeadChip TCGA samples of each cancer type which were not used before (except KIRP which only had one batch) (see Supplementary Table S6). The set of cancer differentially methylated genes was rederived which resulted in 4895 cDMGs including 86.54% of previously identified cDMGs. Then the process of identification age-associated cancer markers was performed following the same method as mentioned above. Finally, 36 genes were regained as age-associated cancer markers by the new ACWN. The results showed that 25 of total 28 markers identified in the experiment dataset were shared with the validation dataset (except for SLC31A1, SLC19A3 and PHYHD1). Therefore 25 shared markers were regarded as stable cancer candidate biomarkers for further analysis. (Table 2, Fig. 6).

*ID4* is a marker specific for BRCA, and this gene exhibits an HH pattern. This gene is a member of the inhibitor of DNA binding protein family, which is a dominant negative regulator of basic helix loop helix transcription factors and plays dominant roles in cancer cells. The methylation level of the tumor suppressor gene *ID4* increased significantly in invasive carcinoma compared with normal breast tissue^44^. In a recent study, Chaudhary *et al.* demonstrated that *ID4* exhibited a novel role of promoting cellular senescence in prostate cancer cells^45^. The protein encoded by *HAND1* is a member of the basic helix-loop-helix family of transcription factors. In the motif analysis of tumor-specific methylated regions in small cell lung cancer (SCLC), Pfeifer *et al.* found that these regions were enriched in the binding site for the neural cell fate-specifying transcription factor *HAND1*^46^. In addition, they also observed increased methylation at the *HAND1* promoter in SCLC lines and primary tumors. *PROX1* is considered to be a tumor suppressor gene in the tumor associated gene (TAG) database^47^. Versmold *et al.* identified *PROX1* as a novel target gene that was hypermethylated and transcriptionally silenced in primary and metastatic breast cancer^48^. *CCL11* is one of several chemokine genes that are overexpressed in ovarian carcinoma. *CCL11* potently stimulates proliferation and migration/invasion of ovarian carcinoma cell lines^49^. Researchers observed age-related increased in *CCL11* expression in plasma and cerebrospinal fluid from healthy human individuals between 20 and 90 years of age^50^. In addition, among the 7 genes identified as age-associated markers in kidney cancer (KIRC and KIRP), 4 genes (*HAND2, FLT4, GDNF*, and *IL22RA1*) were associated with age in kidney tissue^51^.

In conclusion, these results suggest that the method we used to integrate aDMGs and cDMGs into protein-protein interaction networks could assuredly and robustly identify the genes associated with both age and cancer, providing a credible achievement of identification age-associated cancer biomarkers.

### Survival analysis for age-associated cancer markers

To explore whether the markers we identified are considered likely to be of prognostic value, the predicative effect of each marker regarding overall survival (OS) for the corresponding cancer was subsequently assessed by performing survival analysis. The methylation and clinical data of seven cancers were downloaded from TCGA. Notably, we identified three survival-associated methylation markers in kidney renal clear cell carcinoma (KIRC).

We randomly assigned 218 KIRC specimens to a training set (n = 109) and a testing set (n = 109) and found no significant differences in clinical characteristics between these two sets (Supplementary Table S5). Next we only used the training set to examine whether these five markers for KIRC are associated with the survival of patients. In the univariate Cox regression, we found that not only methylation signatures, stage and age also could independently predict patient survival (Table 3). Thus, in the multivariate Cox regression analysis, gene methylation, age, sex and stage were regarded as covariates. As a result, three genes (*HAND2, GDNF* and *ALX1*) were found to be significantly associated with patients survival (p < 0.05) after excluding explanation by other clinical factors such as age, gender and stage, and they were all risky genes (Table 3). From our three-methylation signature, the PI for each patient was calculated as follows:


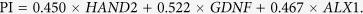


**Figure 7:**
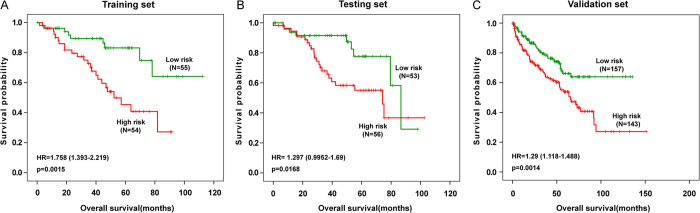
Survival analysis in KIRC. The Kaplan-Meier plots show overall survival probability in high-risk group (Red) and low-risk group (Green). The p value was determined using log-rank test. Overall survival was indicated in months. (**A**) One hundred and nine patients in the training data set. (**B**) One hundred and nine patients in the testing data set. (**C**) Three hundred patients in the independent validation set.

The threshold value for the PI (the median) was −0.25869. Patients in the training set were divided into high-risk and low-risk groups based on their PI values. Consequently, patients with high-risk PI values exhibited a poorer prognosis compared with those with low-risk PI values (Fig. 7A, p = 0.0015). The median survival times of the high-risk and low-risk groups were 36.95 and 45.57 months, respectively. The median ages of the high-risk and low-risk groups were 61.5 and 59 years, respectively, with no significant difference (Student’s t test, p = 0.329).

Next, patients from testing set were classified as high-risk and low-risk based on the same PI formula and threshold. Similar to the findings from the training set, patients in the high-risk group had shorter median survival than patients in the low-risk group (37.05 months versus 46.13 months, Fig. 7B, p = 0.017). Simultaneously, we adopted an independent dataset containing 300 patients from the TCGA Illumina Infinium HumanMethylation450 BeadChip to reconfirm our genes. Likewise, we found that patients in the high-risk group also had poor overall survival patients compared with in the low-risk group (Fig. 7C, p = 0.0014). These results indicate that *HAND2, GDNF* and *ALX1* might be employed as potentially biomarkers not only for early detection of kidney renal clear cell carcinoma but also for predictions of treatment response. A previous study demonstrated that *HAND2*’s promoter undergoes age-associated DNA hypermethylation in epithelial tissues^6^, and this effect may silence *HAND2* in endometrial stromal non-immune cells^14^. *HAND2* may be a contributing factor to cancer risk and serve as a prognosis biomarker for endometrial cancer^52^.

Besides, we would like to explore if we were to pick a random set of genes, and redo the survival analysis, would we find a similar number. As the three genes we identified were both age-associated and prognostic-associated, thus we randomly picked a set of 25 genes from all genes to redo the survival analysis, and then investigated the number of genes which were both age associations and prognostic associations. The process was performed for 10000 times. Of 10000 times, there were 197 times showed that the number of genes which both age associations and prognostic associations was great than or equal to three (p = 0.0197, Supplementary Fig. S6). That means the methodology itself can identify more both age associations and prognostic associations than just picking genes at random.

## Discussion

Aging is one of the largest single risk factors in cancer^53^. In 2014, the World Health Organization (WHO) published the World Cancer Report. This report analyzed the incidence of cancer among different age groups. In contrast to leukemia which primarily occurs in children, the incidence of other cancers increases dramatically with age, demonstrating a high correlation between cancer and aging and suggesting that cancer is a process of aging.

In this study, we performed a large scale genome-wide study of DNA methylation profiles of age and seven cancers using Illumina Infinium HumanMethylation27 BeadChip. We measured the methylation levels of over 27,000 CpGs and provided evidence that DNA methylation is related to aging and carcinogenesis. We demonstrated that the application of an age-associated and cancer-related weighted network can provide biologically meaningful results and that the relevance between age-associated changes in DNA methylation and carcinogenesis was not random.

Age has a profound effect on DNA methylation, and age-associated DNA hypermethylation occurs predominantly at bivalent chromatin promoters. Key developmental genes in promoters are also frequently hypermethylated in cancers^12^. Consistent with this observation, our results demonstrated that hyper-aDMGs were enriched in a variety of developmental processes, thus manifesting a link between aberrant hypermethylation in aging and cancer. Furthermore, we observed more genes (16 genes) exhibiting an HH pattern in the 25 potential cancer markers we identified, indicating that age-associated hypermethylation may play a crucial role in early cancer development. Meanwhile, in the function enrichment analysis, those hypo-aDMGs were significantly enriched in the process of immune response and defense response. However, notably, a number of studies have observed that cell composition is a large source of variability in DNAm data derived from peripheral blood^54^,^55^. Thus the method we adopted for identifying aDMGs many result in some false positives, failure to account for cellular heterogeneity, especially for hypo-aDMGs^14^. Indeed, we observed the age-associated changes in cell subtype composition in our data followed the reference-based procedure of Houseman *et al.*^56^ (Supplementary Fig. S7). It showed that an age-associated increase ratio in the CD8 + T cell and granulocyte, and an age-associated decrease ratio in the CD4 + T cell, NK cell and monocyte, respectively. The interpretation of age-associated changes in whole blood is problematic due to underlying age-associated changes in blood cell subtype proportions. In future study, we will remove the impact of changes in blood cell subtype composition.

In addition to the HH and LL patterns we proposed in this study, two additional patterns (HL pattern and LH pattern) were observed (20 genes indicated in brown in Fig. 5, Supplementary Fig. S8). The genes belonged to these two patterns may not share similar epigenetic mechanism during aging and cancer; however, their methylation levels are also associated with these two processes. We hypothesize that the methylation patterns of these genes in the development of cancer may bypass the mechanism of aging and revert to the initial state of life. The inconformity may also be an exciting phenomenon that displays a different unknown mechanism. This process should be further studied in our future work.

Although aging is a risk factor for cancer, the relationship of aging and cancer is not straightforward. Increased or decreased methylation was noted up to age 70, but these events plateau thereafter during aging in a healthy population. Even a reversal was observed after 80 years of age, e.g., reduced methylation in HH pattern genes, including *HAND2* and *GDNF* (Supplementary Fig. S9). The analysis of demographic data indicates that cancer incidence and mortality plateau around the age of 85 to 90 years^57^. This observation appears to be consistent with our results that suggest that elderly individuals are protected from cancer onset and progression.

There are two major limitations with our work. Many studies have shown that promoter methylation of genes can be proposed as diagnostic and prognostic biomarkers for cancer. Thus we mainly focus on CpG sites in the gene promoters by using the methylation data derived from Illumina Infinium HumanMethylation27 BeadChip to dissect the complex relationship between age and cancer. Since the CpG sites of Illumina Infinium HumanMethylation27 BeadChip are overlapped 14.495 Refseq genes and mainly located in the gene promoters, they may be used to represent the methylation patterns of genes. While, comparing to Illumina Infinium HumanMethylation450 BeadChip, the methylation data from Illumina Infinium HumanMethylation27 BeadChip may partially lead to a bias in the calculation of gene methylation for the loss of a number of CpGs. In the future, as the growth of Illumina Methylation450 BeadChip data, it will be in favor of studying the relationship between age-associated methylation changes and cancer risk. The other limitation in this study is that the aDMGs were identified from a dataset from whole blood, whereas the cDMGs were identified from tissues. To assess the differences between blood and normal tissue, we compared the methylation levels of genes from the two sources regarding coincident stages of age. It was shown that there were minor methylation differences between whole blood and normal tissues. Furthermore, the Pearson correlation coefficients of the methylation levels for all genes were calculated across blood and tissues. The results demonstrated a high correlation between blood and tissues (Supplementary Fig. S10). Despite some limitations for the two sources of data, perhaps, it was feasible that the DNA methylation data across blood and tissues were integrated to identify age-associated cancer markers.

In the post-genomic era, analysis of large biological networks has become an essential task in systems biology research. Accordingly, detecting important function modules contributes to the understanding of the organizational structure of a biological system. Here, given that DNA methylation is involved in the regulation of gene expression, we applied network theory to construct an integrated weighted network by using protein-protein interaction data. Network theoretical approaches of systematically studying diseases have benefitted from numerous gene expression studies; however, DNA methylation data have not been fully utilized. The strategy of integrating network and individual epigenetic features facilitates the identification of more reliable age-associated cancer markers which may commonly function in the development of cancers.

## Materials and Methods

### DNA methylation and gene expression datasets

The DNA methylation profiling with age information used in this study was collected from a public dataset which at NCBI Gene Expression Omnibus under accession number GSE41037^58^. The dataset contains 394 healthy Holland individuals (age range 16–88) that from the control samples of ALS study and SZ study. The Illumina Infinium HumanMethylation27 BeadChip measures bisulfite-conversion-based single-CpG resolution DNA methylation levels at 27,578 different CpG sites within 5′ promoter regions of 14.495 well-annotated genes in the human genome. Since the available age dataset is produced by Illumina Infinium HumanMethylation27 BeadChip, our study is limited to gene promoters.

In this study, we used seven cancers including breast invasive carcinoma (BRCA), colon adenocarcinoma (COAD), kidney renal clear cell carcinoma (KIRC), kidney renal papillary cell carcinoma (KIRP), lung adenocarcinoma (LUAD), lung squamous cell carcinoma (LUSC) and rectum adenocarcinoma (READ) (see Supplementary Table S6). They were collected from The Cancer Genome Atlas (TCGA). DNA methylation levels are also assayed by Illumina Infinium Human Methylation27 BeadChip. Similar to other microarray experiments, methylation data is susceptible to various technical artifacts, particularly batch effects^59^. Consequently, for each cancer, in the experiment dataset, we downloaded methylation data of tumor and normal samples as well as their matched gene expression data only from the same batch that containing the maximum samples. In order to achieve reliable results, we applied our method to a validation dataset. The validation dataset was comprised of all the other TCGA 27 K samples which were not used in the experiment dataset. As there was only one batch for KIRP in TCGA 27 K methylation data, we used the same samples for KIRP in the validation dataset. To reduce the batch effects as possible, we used ComBat in *sva* R package^60^ to process the data before assigning DNA methylation level to gene.

### Preprocessing of methylation data

The HumanMethylation27 BeadChip primarily represents specific CpGs located near 14.495 gene promoter regions. It is likely that multiple CpG sites were mapped to the same gene. In some cases, significant differences in the methylation levels of these CpGs were noted; this difference remained unreasonable even when we used the mean value as the methylation level of gene. Therefore, we utilized the following preprocessed rules to remove such genes. Zhang *et al.* considered that the majority of CpG sites were either hypermethylated or hypomethylated (methylation levels were consistently higher or lower than 0.5, respectively)^29^. Thus, in one sample, if the methylation levels of all CpGs in gene *A* were greater than (or under) 0.5, we considered that the CpG sites in gene *A* had a consistent pattern in this sample. If the ratio of samples with consistent patterns to total samples in gene *A* was greater than a threshold, we calculated the average methylation level of all CpGs as the methylation level of gene *A*. Genes that failed to meet the criterion were excluded from further analysis. We compared different ratios of samples with consistent patterns (60, 70, 80, 90 and 100%; see Supplementary Fig. S11) and made a compromise choice to retain more genes and to improve the accuracy of gene methylation level. As a result, we selected 80% as the threshold and excluded approximately 1,600 genes and their corresponding CpG sites in each dataset including both seven cancer and normal datasets from TCGA as well as an age dataset from GEO. In the subsequent work, we calculated the average values of all remained CpGs within a gene as the methylation level of gene and used this information for analysis.

### Identification of age -associated differentially methylated genes

The genes exhibit age-associated dynamic DNA methylation changes are defined as age-associated differentially methylated genes (aDMGs). To identify the DNA methylation changes associated with age, we fitted a separate linear regression model for each gene, respectively. The significance of correlation was evaluated by perturbing the samples and recalculating the statistics. For each gene we performed 1000 times’ random perturbation and set the threshold of permutation test p value was 0.01.

### Identification of differentially methylated genes in cancer

Here we used the software “QDMR”^30^ developed by Zhang *et al.* to identify differentially methylated genes in cancer (cDMGs) based on the entropy of genes. For each gene in one cancer, we calculated the entropy value of cancer samples and normal samples. The smaller entropy value indicated that greater differences between cancer and normal. In order to explore whether the differentially methylated genes would be affected by age, we compared the age range of cancer samples and normal samples. For almost all cancers (except READ), age was no significant differences in the identification of differentially methylated genes (Wilcoxon rank test, p = 0.836(BRCA); p = 0.9226(COAD); p = 0.5581(KIRC); p = 00.4313(KIRP); p = 1(LUAD); p = 0.589(LUSC); p = 0.00283(READ)). The difference may be caused by a small sample size and a small range of age in normal tissues. Actually, there were not a lot of differences though the p value was less than 0.05. Thus, the final 4452 differentially methylated gene set we used was the union set of differentially methylated genes of seven cancers, including 1691 DMGs in BRCA, 1817 DMGs in COAD, 1203 DMGs in KIRC, 1077DMGs in KIRP, 1658 DMGs in LUAD, 1869 DMGs in LUSC and 1983 DMGs in READ.

### Network weighting and randomization

In this study, a human protein interaction network (PPI) was used as the background network^26^ for further analysis. The network was integrated from five databases, including HPRD, IntAct, DIP, MINT and BIND. An age-associated weighted network (ASWN) was firstly extracted by mapping aDMGs to the background network. The aDMGs were used as network nodes, and the interaction between aDMG pairs served as network edges. Then, the weight of edges in ASWN was determined by Pearson correlation coefficients of the methylation value for each aDMG pair. Next, the methylation data of each pair was perturbed 1000 times, and the correlation coefficients were calculated. All edges for which true methylation correlation coefficients were not within the top 0.5 percent or bottom 0.5 percent of random coefficients were removed from the ASWN (permutation test, p < 0.01).

To elucidate that the age-associated and cancer-related weighted network (ACWN) had biological significances, random subnetworks were generated by random sampling the same number of nodes as noted in ACWN and extracting all of the connections of these sampled nodes from PPI. Then, this process was repeated 1000 times.

### Calculation of network topological features

The topological features of a network provides a quantified method to describe and compare networks. In this study, we used degree and clustering coefficient which are two of the most common topological features to compare the network we constructed and random networks.

### Degree

The degree of a node refers to the number of its neighbor nodes, or the number of edges linked with the node. The degree of a network is defined as average degree of all the nodes which depend on the number of total links and nodes in network. Degree distribution means the distribution of node degrees. In the complex biological networks, degree distribution usually follows a power law that suggesting most nodes have small degrees and only a few nodes have large degrees. Thus they are called as scale-free networks for power law has no fixed pattern which is different from Poisson and exponential distributions.

### Clustering coefficient

Clustering coefficient is specially used to measure of the clustering of network nodes. For a node i, the clustering coefficient *C*_*i*_ is defined as


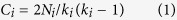


Where 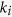 represents the degree of node 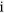, 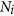 represents the number of all the connections between neighbors of node 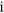. The clustering coefficient of network is defined as the average value of clustering coefficients of all the nodes. The network with a high average clustering coefficient trends to be found a structure of module.

### Survival analysis

The patients of KIRC were randomly assigned to a training set and a testing set. Two sample sets were required to have the same size. The splitting strategy was an advantage over cross-validation, because there was no overlap between the two sample sets. We aimed to identify clinically significant prognostic methylation signatures from the training set and tested them using the corresponding internal testing set and external independent validation set. Firstly, genes were performed Z-score transformation in the training set, then univariate Cox regression analysis was performed to evaluate the association between survival and methylation levels of each gene as well as other clinical factors. As clinical factors were also associated with patient survival, multivariate Cox regression analysis was used to evaluate the independent contribution of each gene to prognostic. Thus, in the multivariate cox regression analysis, the gene methylation, age, sex and stage were used as covariates. Risky genes were defined as hazards ratio > 1, and those with hazards ratio < 1 were defined as protective genes. We assigned each patient a prognostic index (PI) according to a linear combination of the methylation levels of genes which significantly associated with survival weighted by the regression coefficients from multivariate Cox regression aforementioned. The prognostic index for each patient was calculated as follows:


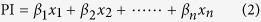


Where 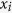 was the Z-score normalized methylation level of gene 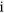, and 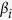 was the regression coefficient of gene 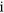 in the multivariate Cox regression. We divided patients in training set into high-risk group and low-risk group by using the median PI as a cut-off point. The differences of age between high-risk and low-risk groups were analyzed by Student’s t test. Kaplan-Meier method was used to estimate overall survival and log-rank test was used to investigate whether different groups had a significantly different survival.

Then we used two ways of validation: internal validation versus external validation. The testing set was used as internal validation and the TCGA Illumina Infinium HumanMethylation450 BeadChip dataset for KIRC patients (n = 300) was used as an external independent validation. In the independent validation set, there is no patient overlapped with patients in the training set or testing set. In TCGA Illumina Infinium HumanMethylation450 BeadChip data, we only selected the CpGs concordant with Illumina Infinium HumanMethylation27 BeadChip and obtained gene methylation level by using the method aforementioned. The regression coefficients and the threshold of PI derived from the training set were directly applied to the methylation data of the testing set and the independent validation set, and then the patients in each set were classified to high-risk group and low-risk group, respectively. The evaluation of survival time and the comparison of differences between two groups were the same as that in the training set.

## Additional Information

**How to cite this article**: Wang, Y. *et al.* The identification of age-associated cancer markers by an integrative analysis of dynamic DNA methylation changes. *Sci. Rep.* **6**, 22722; doi: 10.1038/srep22722 (2016).

## Supplementary Material

Supplementary Information

Supplymentary Table S1

Supplymentary Table S2

Supplymentary Table S3

Supplymentary Table S4

## Figures and Tables

**Table 1:** Biological_Process enrichment analysis for genes in the modules.

Term	Count	P-value	Bonferroni
GO:0048731~system development	69	9.49e-10	1.69e-06
GO:0048513~organ development	57	1.54e-09	2.76e-06
GO:0048856~anatomical structure development	70	1.17e-08	2.10e-05
GO:0007275~multicellular organismal development	75	2.93e-08	5.23e-05
GO:0032502~developmental process	79	6.26e-08	1.12e-04
GO:0010628~positive regulation of gene expression	27	1.99e-07	3.56e-04
GO:0009888~tissue development	29	2.27e-07	4.05e-04
GO:0009891~positive regulation of biosynthetic process	29	5.55e-07	9.90e-04
GO:0051254~positive regulation of RNA metabolic process	23	1.30e-06	2.32e-03
GO:0048522~positive regulation of cellular process	52	1.45e-06	2.58e-03
GO:0045941~positive regulation of transcription	25	1.50e-06	2.67e-03
GO:0009611~response to wounding	24	1.81e-06	3.22e-03
GO:0007399~nervous system development	36	3.67e-06	6.52e-03
GO:0006954~inflammatory response	18	3.84e-06	6.83e-03
GO:0048518~positive regulation of biological process	54	4.81e-06	8.55e-03
GO:0009605~response to external stimulus	31	1.37e-05	2.41e-02

**Table 2:** Age-associated cancer markers in various cancers.

Symbol	ID	Markers in cancer	Pattern
*HAND2*	9464	KIRC	HH
*GDNF*	2668	KIRC	HH
*ALX1*	8092	KIRC	HH
*UTF1*	8433	LUSC	HH
*FLT4*	2324	KIRP	HH
*KDR*	3791	COAD	HH
*HAND1*	9421	LUAD	HH
*ID4*	3400	BRCA	HH
*PROX1*	5629	BRCA	HH
*SOX11*	6664	READ	HH
*MYO3A*	53904	READ	HH
*NCAM1*	4684	COAD, READ	HH
*EVC*	2121	COAD, READ	HH
*NRG1*	3084	COAD, READ	HH
*GFRA1*	2674	LUAD	HH
*SORCS1*	114815	LUSC, COAD, READ	HH
*SLC17A2*	10246	KIRC, KIRP	LL
*IL22RA1*	58985	KIRP	LL
*CCL11*	6356	BRCA	LL
*CYP2D6*	1565	READ	LL
*NPC1L1*	29881	COAD	LL
*SERPINA4*	52567	COAD	LL
*CPXCR1*	53336	COAD	LL
*ITIH3*	3699	KIRC	LL
*MRTO4*	51154	LUSC	LL

**Table 3:** Univariate and multivariate survival analyses for KIRC patients in the training set.

Variable	Univariate analysis			Multivariate analysis		
	HR(95%CI)	Regression coefficient	p-value	HR(95%CI)	Regression coefficient	p-value
Stage	1.594(1.183–2.148)	0.467	**2.00 × 10**^**−3**^	—	—	—
Age	1.032(1.002–1.063)	0.031	**3.90 × 10**^**−2**^	—	—	—
Sex	1.285(0.631–2.619)	0.251	4.89 × 10^**−**1^	—	—	—
*HAND2*	1.789(1.328–2.411)	0.582	**1.33 × 10**^**−4**^	1.568(1.120–2.195)	0.450	**8.82 × 10**^**−3**^
*GDNF*	1.919(1.466–2.512)	0.652	**2.08 × 10**^**−6**^	1.686 (1.212–2.345)	0.522	**1.93 × 10**^**−3**^
*ALX1*	1.826(1.299–2.566)	0.602	**5.31 × 10**^**−6**^	1.595(1.117–2.276)	0.467	**1.02 × 10**^**−2**^
