# Supplementary material for: The identification of age-associated cancer markers by an integrative analysis of dynamic DNA methylation changes
Source: Sci Rep. 2016 Mar 7;6:22722. doi: 10.1038/srep22722 (PMC4779991; doi:10.1038/srep22722)
Supplement: Supplementary Information [file srep22722-s1.pdf]

The identification of age-associated cancer markers by an integrative analysis of dynamic DNA methylation changes

Yihan Wang[1*], Jingyu Zhang[2*], Xingjun Xiao[3*], Hongbo Liu[1], Fang Wang[1], Song Li[1], Yanhua Wen[1], Yanjun Wei[1], Jianzhong Su[1], Yunming Zhang[2], Yan Zhang[1]

[1]College of Bioinformatics Science and Technology, Harbin Medical University, Harbin, 150081, China.
[2]Department of Gerontology, The First Affiliated Hospital of Harbin Medical University, Harbin 150001, China.
[3]Department of Neurology, The Second Affiliated Hospital of Harbin Medical University, 246 Xuefu Road, Harbin 150086, China

Supplementary Figures

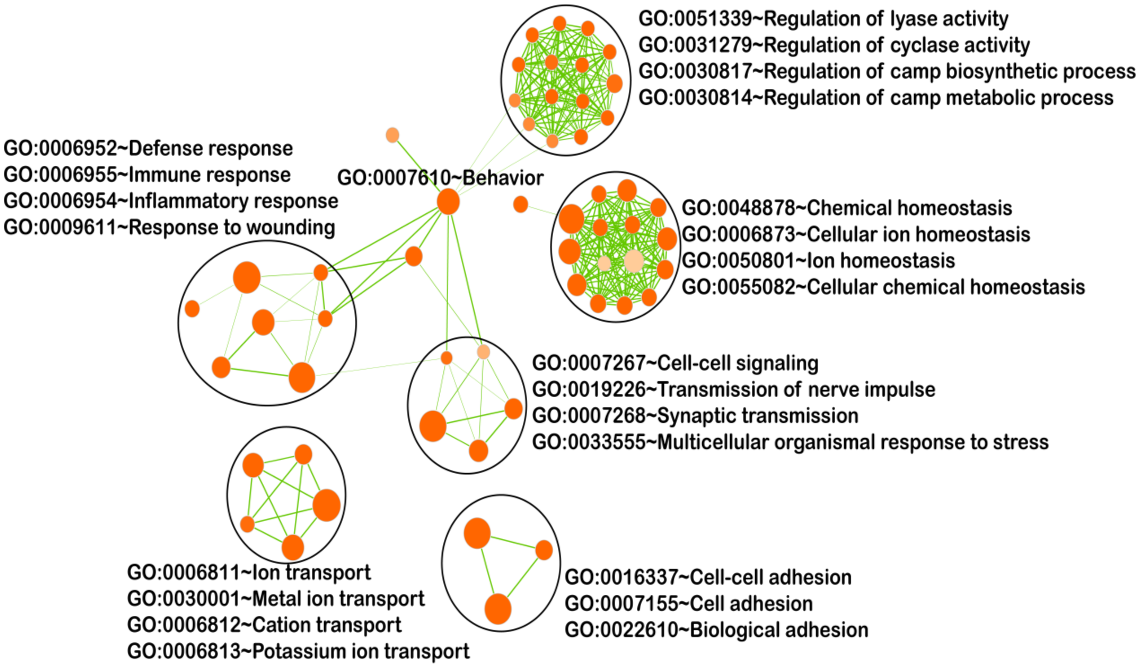

Supplementary Fig. S1 GO biological process enrichment analysis for cDMGs by DAVID. The size of a node represented the number of enriched genes in this term, and the color of a node represented the Bonferroni adjusted p-value of this GO term.

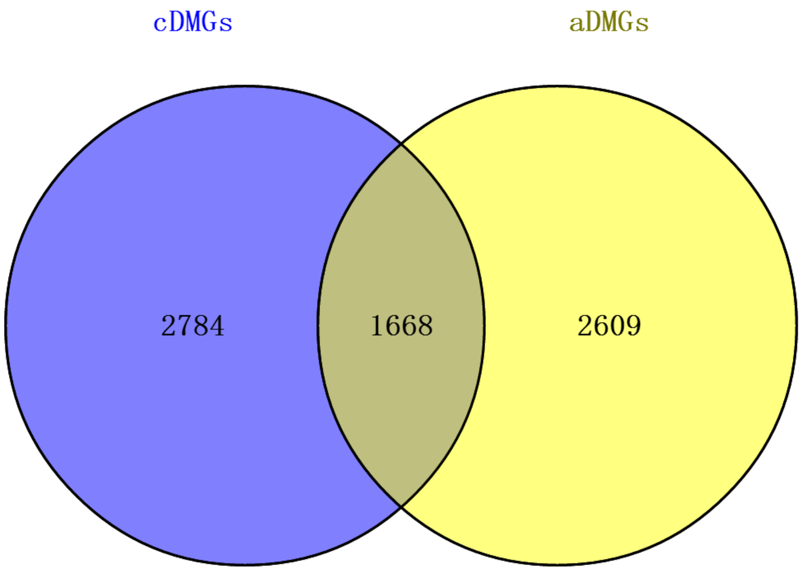

Supplementary Fig. S2 The overlap of differentially methylated genes in cancer (cDMGs) and differentially methylated genes in aging (aDMGs).

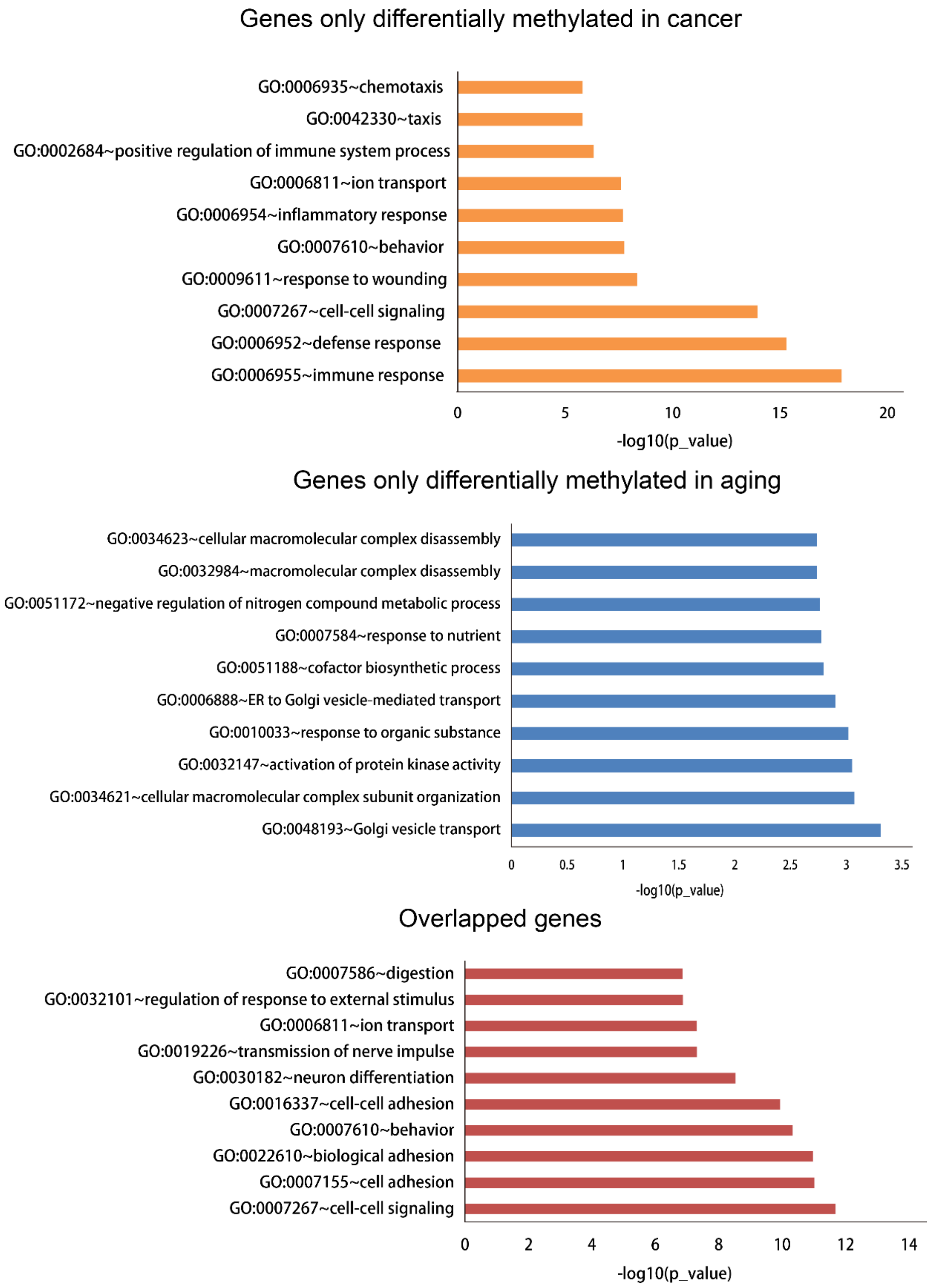

Supplementary Fig. S3 GO biological process enrichment analysis for genes only differentially methylated in cancer, genes only differentially methylated in aging and overlapped genes

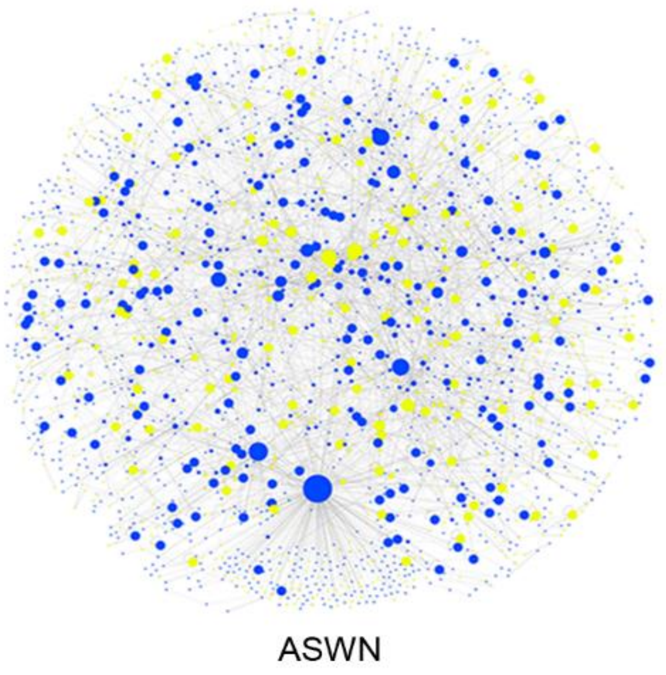

Supplementary Fig. S4 An age-associated weighted network. In the ASWN, yellow nodes represent the genes are hyper-aDMGs and blue nodes represent the genes are hypo-aDMGs. The size of a node represents its degree in the network.

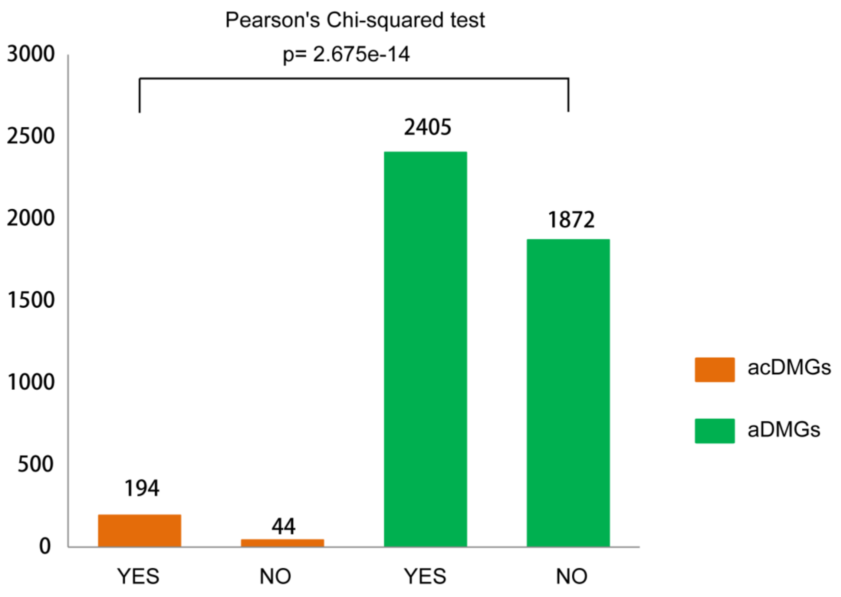

Supplementary Fig. S5 Pearson's Chi-squared test for numbers of acDMGs and aDMGs in or not in the two patterns described by us. That hyper-aDMGs in the

module are more tended to be in low methylation pattern and hypo-aDMGs are more tend to be in high methylation pattern. “YES” represents genes are in one of the two patterns, and “NO” represents genes are not in any of the two patterns.

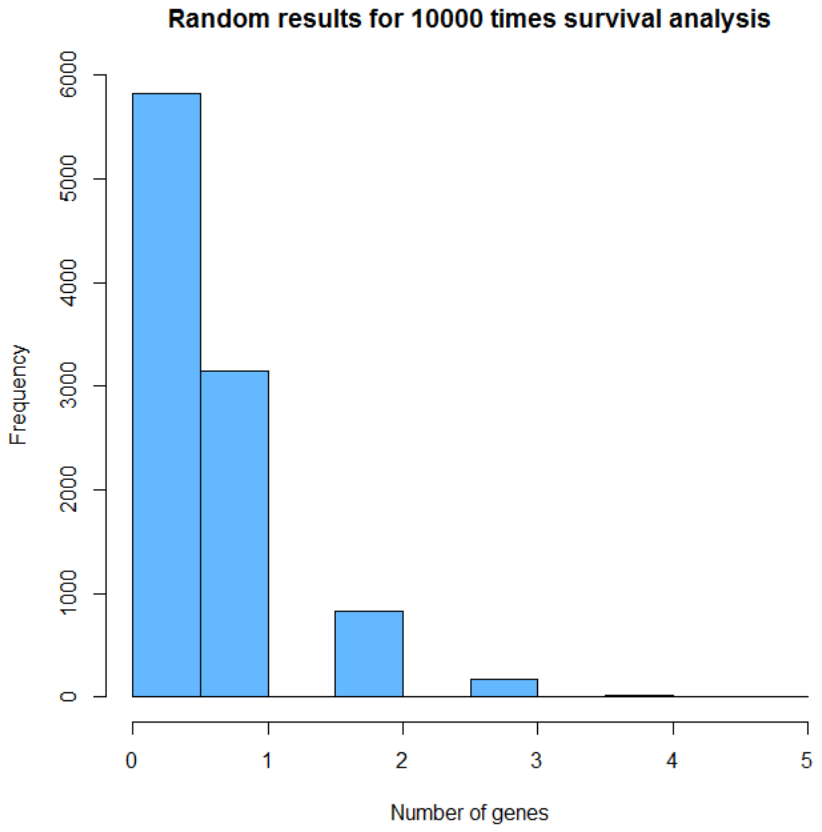

Supplementary Fig. S6 The result of 10000 random processes of survival analysis. The number of genes which both age-associated and prognostic-associated in a survival analysis (x-axis) and frequency of each number (y-axis).

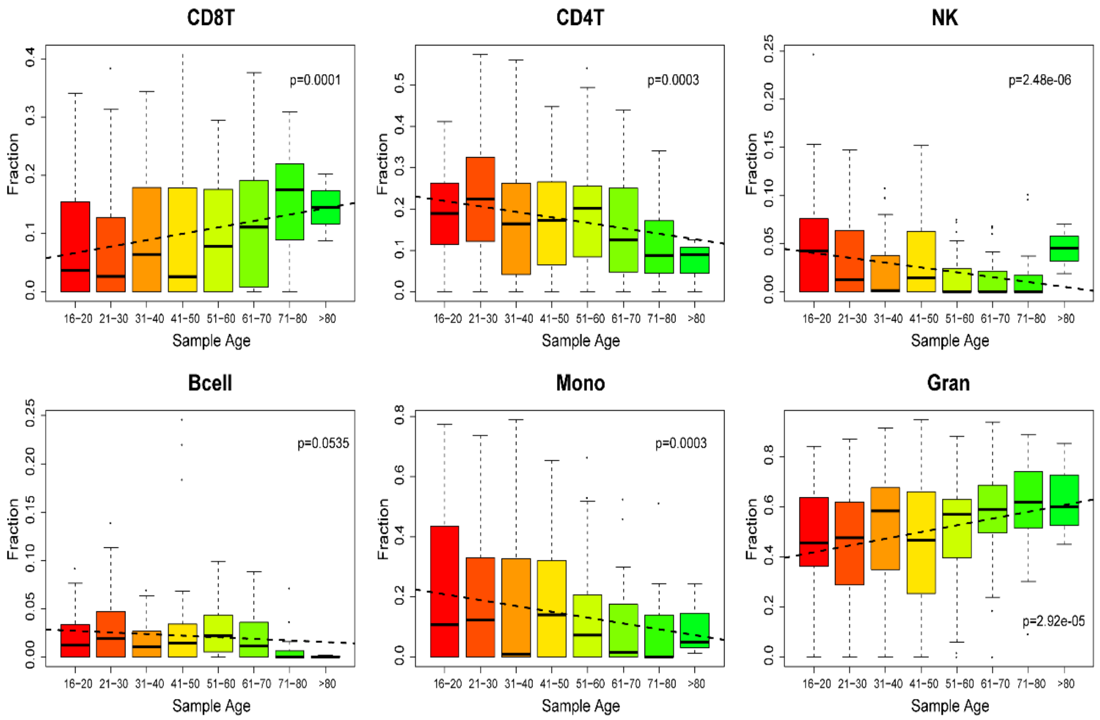

Supplementary Fig. S7 Boxplots showing the change of methylation in cell proportions in our data. Each plot represents one blood cell subtype.A linear regression line and P-value has been added to each plot.

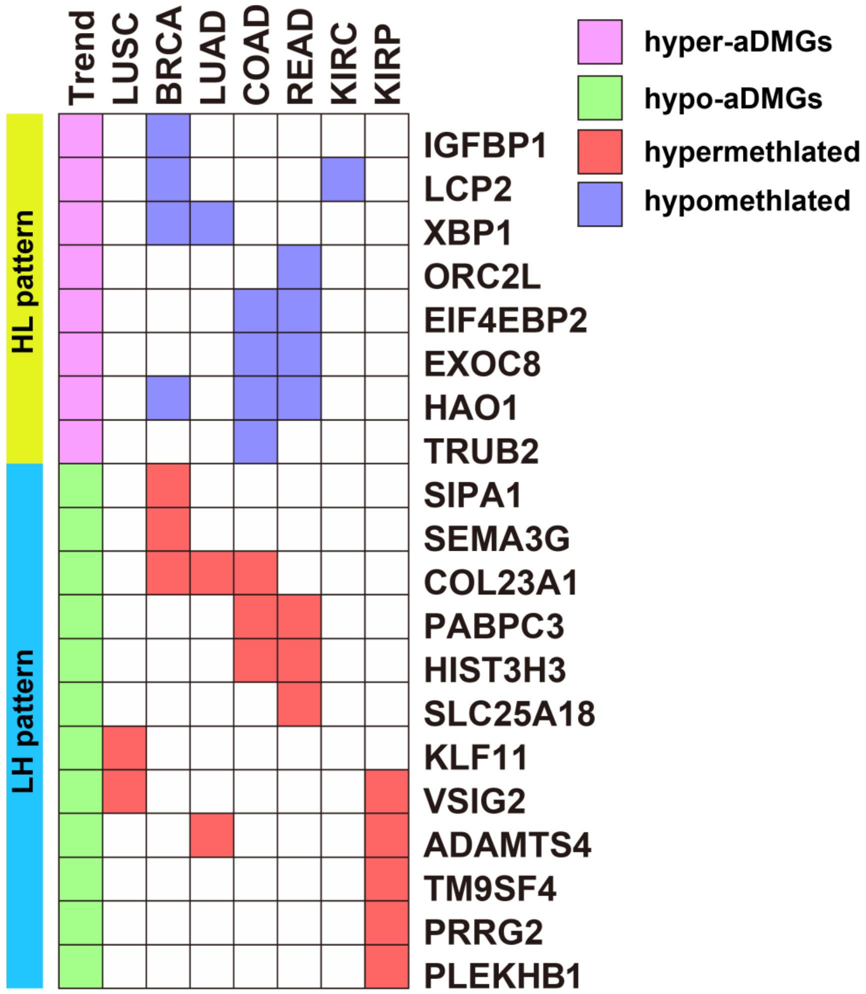

Supplementary Fig. S8 Two additional patterns (HL pattern and LH pattern). In HL pattern, the methylation level of a gene is increasing with aging and lower in cancer than normal. While in LH pattern, the methylation level of a gene is decreasing with aging and higher in cancer than normal.

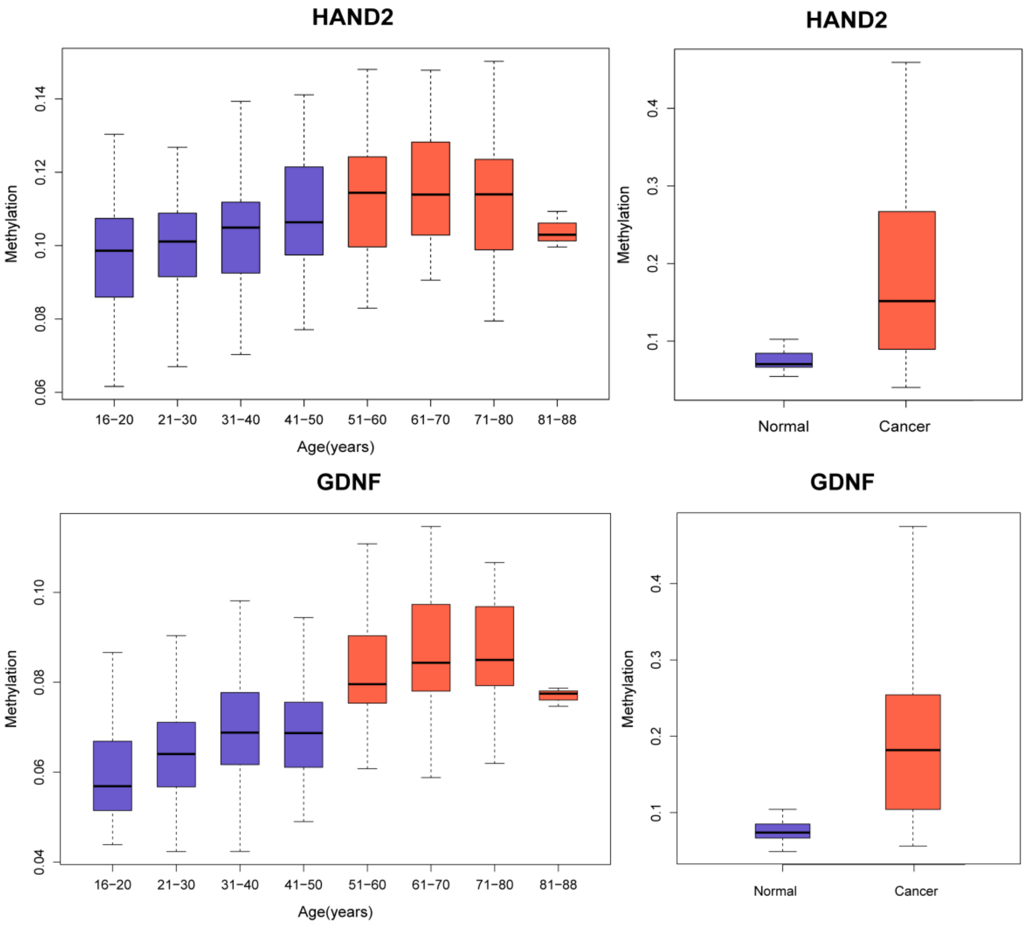

Supplementary Fig. S9 Comparision of methylation distribution of gene HAND2 and GDNF in age samples and TCGA KIRC samples.

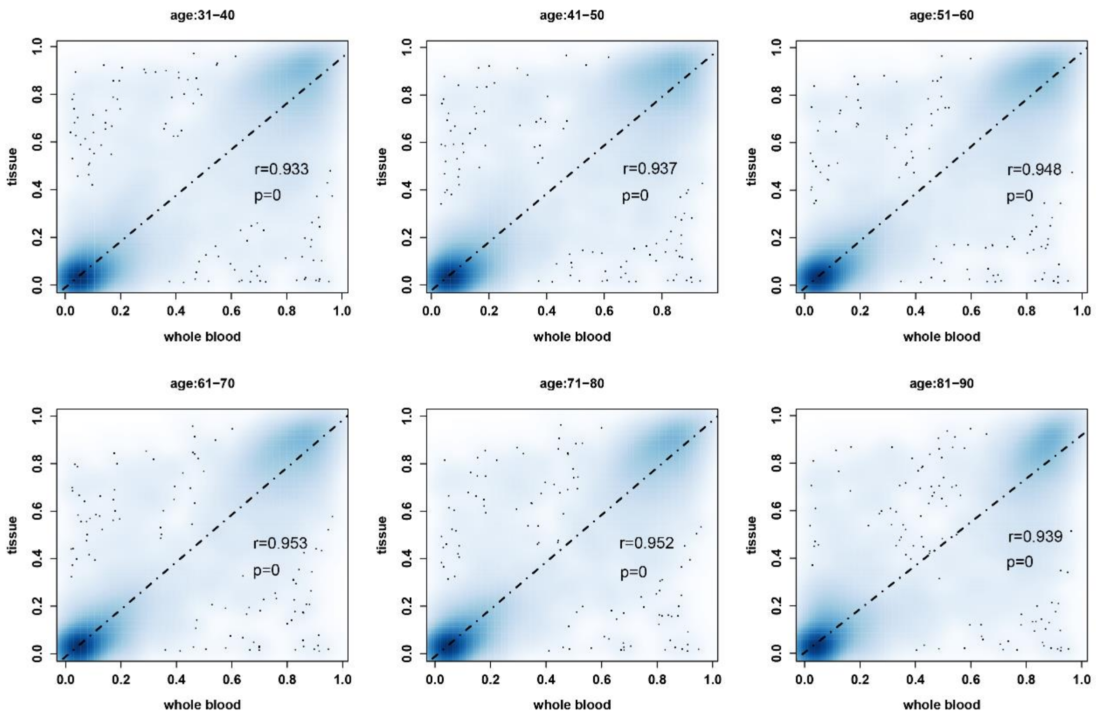

Supplementary Fig. S10 Comparision of gene methylation between whole blood and normal tissues. We compared the methylation levels of genes between a whole blood dataset and TCGA seven normal tissues datasets for different stage of age. “r” represented the Pearson correlation coefficient.

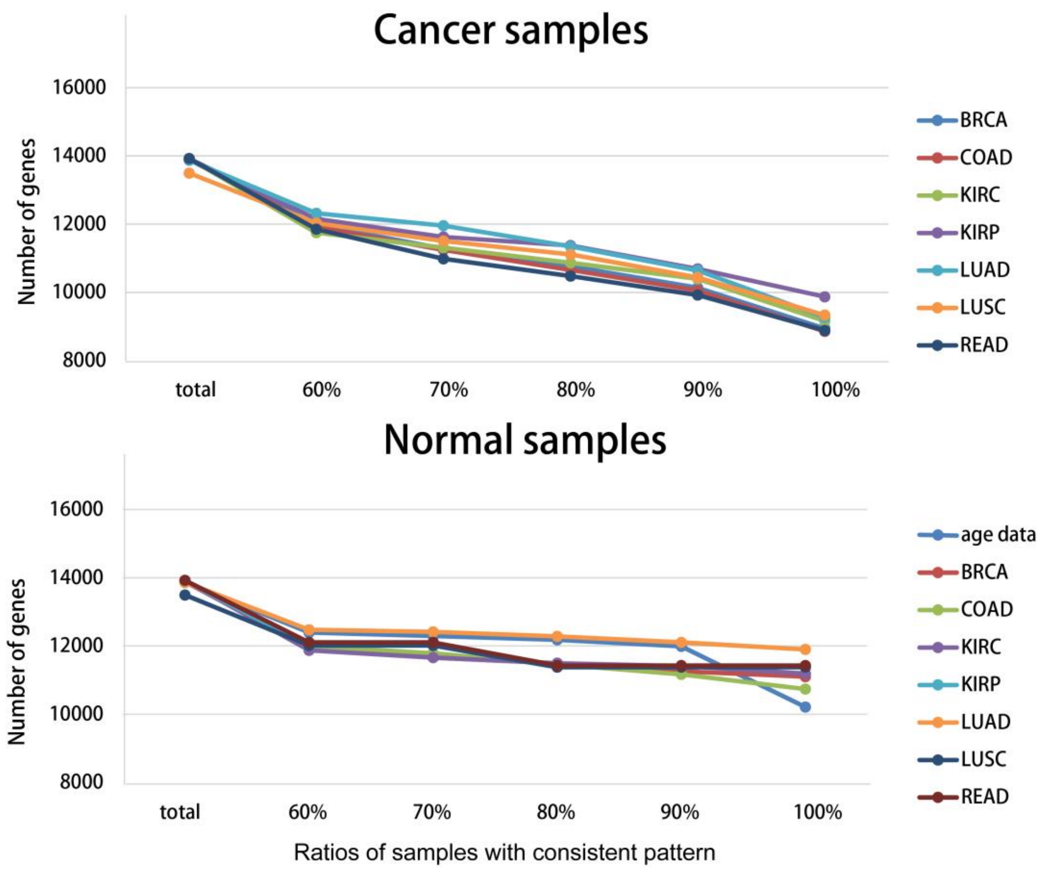

Supplementary Fig. S11 Comparison of different ratios of samples with consistent pattern.

Supplementary Tables

Table S5 Clinicopathologic characteristics of patients with KIRC in TCGA 27K data (N=218)

Characteristics	Number of patients			*p*-value
	All patients N=218	Training set N=109	Test set N=109	
Stage				1[a]
I	115	57	57	
II	27	14	13	
III	52	26	26	
IV	25	12	13	
Age				0.68[b]
Mean±SD	59.55±12.51	60.01±12.21	59.31±12.69	
Range	33-86	34-86	33-85	
Sex				1[a]
Female	77	39	38	
Male	141	70	71	
Survival(month)				0.93[b]
Mean±SD	41.72±24.91	41.55±25.76	41.89±24.15	
Range	0.07-112.57	0.5-112.57	0.07-102.47	
State				1[a]
Living	153	76	77	
Death	65	33	32	

[a] *p*-values were determined using Fisher's exact test.

[b] *p*-values were determined using Student's t- test.

Table S6 Table S6 The number of TCGA HumanMethylation27 samples used in this study.

	Experiment dataset		Validation dataset	
	cancer	normal	cancer	normal
BRCA	44	20	272	7
COAD	31	17	135	20
KIRC	34	25	184	174
KIRP	16	5	16	5
LUAD	35	17	91	7
LUSC	36	27	98	27
READ	31	4	35	5
